# Phosphoinositide 3-Kinase/Akt Signaling and Redox Metabolism in Cancer

**DOI:** 10.3389/fonc.2018.00160

**Published:** 2018-05-15

**Authors:** Nikos Koundouros, George Poulogiannis

**Affiliations:** ^1^Department of Cancer Biology, Institute of Cancer Research, London, United Kingdom; ^2^Division of Computational and Systems Medicine, Department of Surgery and Cancer, Imperial College London, London, United Kingdom

**Keywords:** reactive oxygen species, cancer, phosphoinositide 3-kinase/Akt signaling, oxidative stress, metabolism

## Abstract

Metabolic rewiring and the consequent production of reactive oxygen species (ROS) are necessary to promote tumorigenesis. At the nexus of these cellular processes is the aberrant regulation of oncogenic signaling cascades such as the phosphoinositide 3-kinase and AKT (PI3K/Akt) pathway, which is one of the most frequently dysregulated pathways in cancer. In this review, we examine the regulation of ROS metabolism in the context of PI3K-driven tumors with particular emphasis on four main areas of research. (1) Stimulation of ROS production through direct modulation of mitochondrial bioenergetics, activation of NADPH oxidases (NOXs), and metabolic byproducts associated with hyperactive PI3K/Akt signaling. (2) The induction of pro-tumorigenic signaling cascades by ROS as a consequence of phosphatase and tensin homolog and receptor tyrosine phosphatase redox-dependent inactivation. (3) The mechanisms through which PI3K/Akt activation confers a selective advantage to cancer cells by maintaining redox homeostasis. (4) Opportunities for therapeutically exploiting redox metabolism in *PIK3CA* mutant tumors and the potential for implementing novel combinatorial therapies to suppress tumor growth and overcome drug resistance. Further research focusing on the multi-faceted interactions between PI3K/Akt signaling and ROS metabolism will undoubtedly contribute to novel insights into the extensive pro-oncogenic effects of this pathway, and the identification of exploitable vulnerabilities for the treatment of hyperactive PI3K/Akt tumors.

## Introduction

Tumorigenesis is a multi-step process involving the complex interplay of several biological processes that are highly dependent on the activation of pro-proliferative and pro-survival signaling cascades, accumulation of genetic aberrations, and adaptation to various microenvironmental stress conditions (1, 2). Underpinning these tumorigenic processes is the ability of cancer cells to alter their metabolism to promote nutrient synthesis or scavenging to support their high proliferation demands (3, 4). A major consequence of such extensive metabolic rewiring is the production of reactive oxygen species (ROS), which include both free radical molecules such as superoxide $\left({\text{O}}_{2}^{•-}\right)$ and hydroxyl radicals (^·^OH), as well as non-free radical species, of which hydrogen peroxide (H_2_O_2_) is among the most prominent (5). Although elevated ROS levels can have detrimental consequences on cell viability through extensive damage of proteins, DNA, and organelles, the concomitant increase in antioxidant and detoxification capacities in cancer cells allows for redox homeostasis, thus generating a tightly regulated system whereby ROS can promote tumorigenesis and cancer progression (6, 7). Specifically, there is renewed interest in examining the implications of redox balance on cancer cell proliferation and survival through the regulation of key signaling cascades such as the phosphoinositide 3-kinase and AKT (PI3K/Akt) pathway, which is of particular relevance as it controls many hallmarks of cancer (1, 8, 9). In this review, we will examine the emerging relationship between redox homeostasis and PI3K/Akt signaling, and discuss how their cross-regulation may promote cancer pathogenesis. In addition, we present and look with optimism opportunities toward a future therapeutic exploitation of redox homeostasis in tumors with enhanced PI3K/Akt activation.

## The PI3K/AKT Pathway at a Glance

The intricacies of PI3K/Akt signaling have been extensively reviewed previously (10–12), and will only be briefly introduced here. Activation of receptor tyrosine kinases (RTKs) or G-protein-coupled receptors facilitate the recruitment of class I PI3Ks which phosphorylate phosphatidylinositol-(4,5) bisphosphate (PIP_2_) to phosphatidylinositol (3,4,5)-trisphosphate (PIP_3_). Oncogenic mutations in *PIK3CA*—the gene encoding the class I PI3K catalytic subunit p110α—are found in approximately one-third of human cancers and 40% of breast cancers (13, 14). The accumulation of PIP_3_ allows for the localization of AKT to the plasma membrane and its subsequent activation following phosphorylation on serine 473 and threonine 308 by mTORC2 and PDK1, respectively. Negative regulation of the PI3K/Akt pathway is largely mediated by the phosphatase and tensin homolog (PTEN) through dephosphorylation of PIP_3_ to PIP_2_. AKT is an important downstream effector of oncogenic PI3K signaling and regulates several pathways, including inhibition of apoptosis, stimulation of mTORC1-dependent cell growth, and modulation of cellular metabolism (11). Glucose and glutamine are considered as essential nutrients for cancer cell proliferation, and PI3K/Akt signaling has been shown to regulate the cellular metabolism of both nutrients. Specifically, AKT may directly increase glucose uptake through activation of the glucose transport receptor GLUT1, and stimulate glycolysis by phosphorylating hexokinase 2 (11, 15). Moreover, activating mutations in *PIK3CA* render colorectal cancer cells more dependent on glutamine anaplerosis to replenish TCA cycle intermediates through upregulation of glutamate pyruvate transaminase 2, and glutamine deprivation significantly reduces the proliferation of *PIK3CA* mutant, but not wild type, cancer cells (16). Later work has also demonstrated the importance of Akt-independent signaling cascades associated with PI3K activation, with a particular focus on serum and glucocorticoid-regulated kinases (SGKs) (17).

## Regulation of ROS Production by PI3K/AKT Signaling

**Figure 1:**
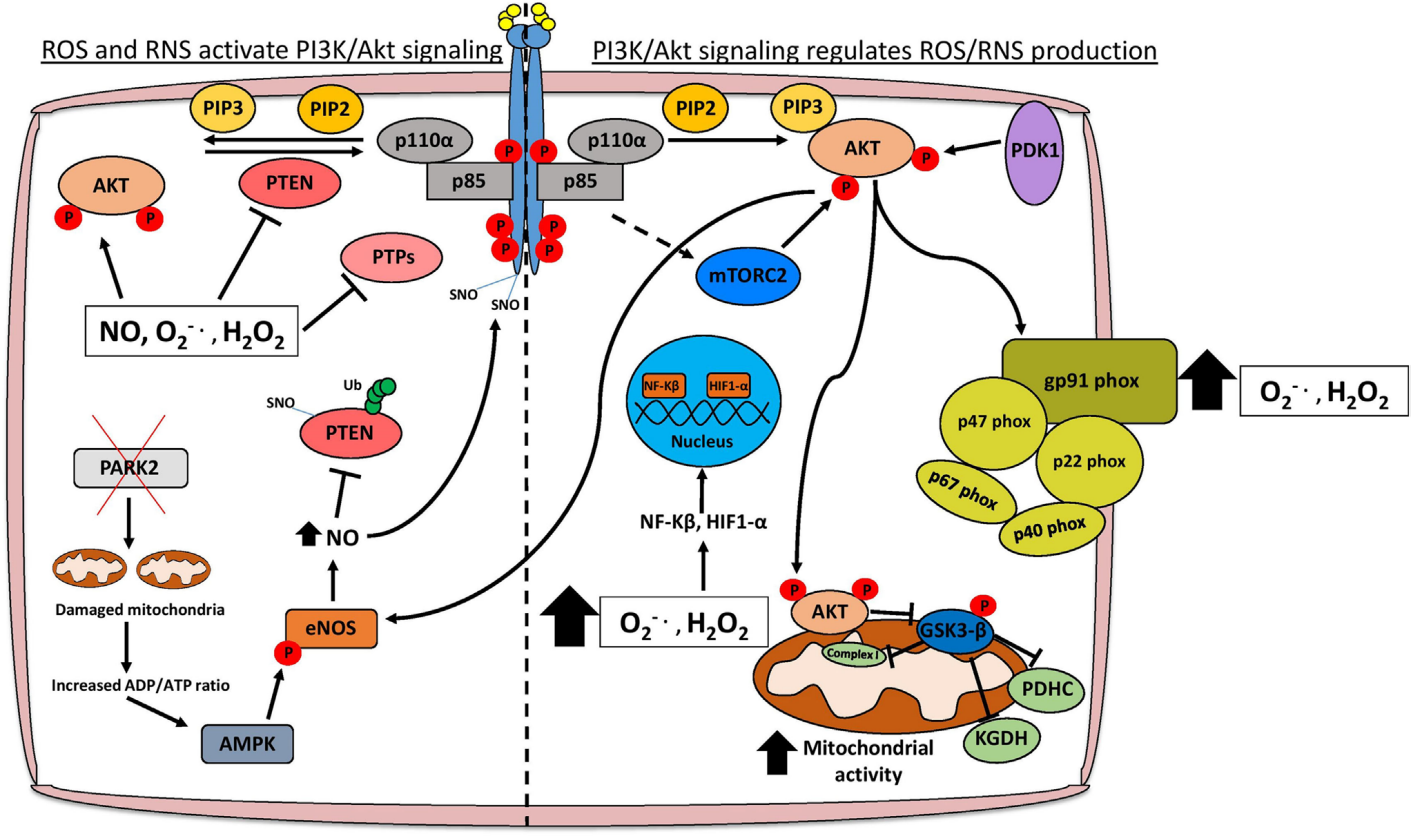
Schematic representation of the interplay between PI3K/Akt signaling and redox stress. Activation of PI3K/Akt signaling occurs following stimulation of receptor tyrosine kinases (RTKs) or G-protein-coupled receptors. AKT is an important oncogenic effector of PI3K signaling and positively regulates reactive oxygen/nitrogen species (ROS/RNS) production through direct modulation of mitochondrial bioenergetics and activation of NADPH oxidases. Cellular ROS levels can also potentiate the PI3K cascade by directly activating AKT, stimulating RTKs through SNO, or through inhibition of various tumor suppressor proteins such as PTEN and PTPs. Moreover, elevated ROS levels promote the nuclear translocation of NF-κβ and HIF1-α which transcriptionally regulate several genes involved in cancer cell growth, proliferation, and survival. Abbreviations: PTP, protein tyrosine phosphatase; SNO, S-nitrosylation; ${\text{O}}_{2}^{•-}$, superoxide; H_2_O_2_, hydrogen peroxide; NO, nitric oxide; eNOS, endothelial nitric oxide synthase; Ub, ubiquitin; PDHC, pyruvate dehydrogenase complex; KGDH, alpha-ketoglutarate dehydrogenase; PTEN, phosphatase and tensin homolog; PI3K, phosphoinositide 3-kinase.

Aberrant PI3K/Akt signaling drives many of the molecular mechanisms contributing to increased ROS levels through direct modulation of mitochondrial bioenergetics and activation of NADPH oxidases (NOXs), or indirectly through the production of ROS as a metabolic by-product (Figure 1) (6, 18). Mitochondria are a major source of cellular ROS, and these are largely derived from electron leakage at complexes I and III of the electron transport chain (6, 19). Complex I in particular has been shown to generate ROS through two mechanisms: the first involves reduction of O_2_ by flavin mononucleotide following binding of NADH induced by high NADH/NAD^+^ ratios, and the second is through reverse electron transport whereby excessive NADH is produced following reduction of NAD^+^ from ubiquinol (6, 20). Interestingly, AKT has been shown to translocate to the mitochondrial matrix and inner membrane in a PI3K-dependent manner following IGF-1 stimulation (21). AKT may directly phosphorylate mitochondrial GSK-3β, reducing its activity and thus alleviating the negative regulation imposed on pyruvate dehydrogenase and α-ketoglutarate dehydrogenase complexes, which have been reported to generate superoxide and H_2_O_2_ (19, 22, 23).

The activation of NOXs by PI3K/Akt signaling also contributes to higher ROS levels in cancer cells (Figure 1) (18). There are seven members of the NOX enzyme family (NOX1-5, DUOX1, and DUOX2) that are expressed across several tissue types including the liver, lung, and gastrointestinal tract (24). NOXs are composed of different subunits including p67^phox^, p47^phox^, p40^phox^, p22^phox^, RAC1, RAC2, and NOXO1/NOXA1 which facilitate the localization of the enzyme complex to the cytoplasm or plasma membrane (24). The main function of NOX enzymes was first ascertained in phagocytes as contributing to the respiratory burst through production of superoxide following electron transfer from NADPH to oxygen (25). The activity of these enzymes is also becoming increasingly associated with various hallmarks of cancer including angiogenesis and metastasis (26). The importance of PI3K/Akt signaling in activating NOXs has been demonstrated by specifically ablating NOX activity following treatment with the PI3K inhibitor wortmannin or knockout of *Akt1* (18, 27). In terms of cancer progression, PI3K-mediated activation of NOX is necessary to promote cell migration and chemotaxis in response to stimulants such as hepatocyte growth factor (HGF) and platelet-derived growth factor (PDGF) (28, 29). Migration of lung endothelial cells requires HGF-mediated activation of c-MET, which signals through the PI3K/Akt pathway and results in the accumulation of p47^phox^/Rac1 in lamellipodia and localized production of H_2_O_2_ to the leading edge of the cell (29). Consequently, inhibition of the PI3K/Akt pathway also inhibits the translocation of NOX subunits and reduces ROS, hindering the metastatic potential of lung cancer cells, at least in part, by reducing expression of the metalloprotease MMP9 and the pro-metastatic miRNA miR-21 (29–31). Angiogenesis is necessary for tumor growth especially following metastasis, and NOX isoforms have been implicated in re-vascularization particularly in PI3K/Akt-hyperactive tumors (32). Vascular endothelial growth factor is the most potent stimulant of angiogenesis and can activate NOX isoforms either directly or indirectly through PI3K/Akt induction (32–34). The subsequent production of superoxide and H_2_O_2_ are necessary for the regulation of transcription factors, which promote angiogenesis, including NF-κB, MMPs, COX-2, and HIF-1α (32).

It is well established that hyperactive PI3K/Akt signaling contributes to increased glycolytic rate, and recent studies have identified dependencies on specific metabolic enzymes that, when perturbed, impact glycolytic flux and consequently cancer cell viability (35). Notably, *PIK3CA* mutant cancer cell lines have elevated 2-oxoglutarate dehydrogenase (OGDH) activity, and inhibition of this enzyme leads to reduced tumor growth *in vivo* (35). The sensitivity to OGDH inhibition can be explained by the observed decrease in the NAD^+^/NADH ratio following accumulation of 2-oxoglutarate, thus limiting the available NAD^+^ needed for glycolysis (35). Interestingly, OGDH is a potent source of ROS and this, together with the need to maintain a stable NAD^+^/NADH ratio, suggests that maintaining redox homeostasis is essential for the proliferation of *PIK3CA* mutant tumors (35, 36). Another important ROS-generating metabolic pathway that is regulated by PI3K/Akt signaling is the production of prostaglandins (PGE_2_) from arachidonic acid by the cyclooxegenases COX-1 and COX-2 (37). The peroxidase activity of the COX enzymes results in the generation of superoxide as a by-product (38). Recent clinical studies have also highlighted the importance of PGE_2_ metabolism in PI3K-driven tumors, with *PIK3CA* mutation status being used as a biomarker for aspirin treatment in colorectal cancer (39). Thus, in addition to the direct effects of PI3K/Akt pathway in augmenting ROS levels, several indirect mechanisms associated with the intrinsic metabolic dependencies of *PIK3CA* mutant tumors exist, which could also contribute to the cellular pool of ROS levels.

## Activation of PI3K/AKT and Pro-Tumorigenic Signaling by ROS

Elevated pro-proliferative signaling cascades and inhibition of growth suppressors are necessary for tumorigenesis, and high ROS levels affect both, by potentiating activation of PI3K/Akt signaling mainly through inhibition of phosphatases such as PTEN or direct activation of oncogenes including AKT (40, 41). In breast cancer, exposure to hormones including 17-β estradiol and its derivative 4-OH-E2 lead to a dose-dependent increase in ROS levels and consequent malignant transformation of MCF10A cells *in vitro* and *in vivo* (42). Mechanistically, this is dependent on ROS-mediated hyper-phosphorylation of PI3K and subsequent downstream PDK1-mediated activation of AKT, culminating in the upregulation of cell cycle promoting genes such as *CDK1* and *PCNA* (42). Interestingly, treatment with ROS scavengers such N-acetylcysteine (NAC) or knockdown of *AKT1* rescues the malignant transformation induced by 4-OH-E2 exposure, indicating an important regulatory role of ROS on PI3K and AKT (42). This regulation has also been demonstrated in T-cell acute lymphoblastic leukemia (T-ALL) where interleukin-7 (IL-7) enhances NOX and mitochondrial complex I activity, thus contributing to elevated ROS in T-ALL cells (43). AKT phosphorylation and activation is induced by the IL-7-dependent increase in ROS levels and promotes glucose uptake through upregulation of the GLUT1 receptor (43).

Excessive oxidative stress also activates PI3K/Akt signaling by inhibiting the activity of its negative regulator PTEN, one of the most frequently altered tumor suppressor genes in cancer (44). High ROS levels modulate PTEN through direct oxidation leading to reduced phosphatase activity, or indirectly through phosphorylation, which increases its stability and prevents its recruitment to the membrane (45, 46). For example, H_2_O_2_ mediated oxidation of cysteine residues 124 and 71 on PTEN leads to the reversible formation of disulfide bridges and reduction of its catalytic activity (47, 48). PTEN oxidation and subsequent inactivation can also occur in the absence of H_2_O_2_ treatment. This has been demonstrated in the COX-2- and LOX-5-dependent synthesis of prostaglandins from arachidonic acid in pancreatic cancer cells (49). Oxidized PTEN as a result of arachidonic acid metabolism does not migrate at a lower molecular weight under non-reducing conditions, whereas a migratory shift is seen following the formation of a disulfide bond between Cys-124 and Cys-71 upon H_2_O_2_ treatment (49). As COX-2 expression and activity is regulated by AKT, a model could exist in pancreatic tumors whereby arachidonic acid metabolism perpetuates PI3K/Akt activity through PTEN inactivation, consequently resulting in persistent prostaglandin production and associated inflammation (50, 51).

Oxidative stress can also induce posttranslational modifications, which facilitate PTEN ubiquitylation and subsequent degradation. One such redox-dependent modification, which contributes to PTEN inactivation and enhanced ubiquitin-proteasome degradation is S-nitrosylation (SNO) (52, 53). Mitochondrial dysfunction, which is triggered by loss of the tumor suppressor *PARK2* leads to a reduction in ATP levels and concomitant activation of AMPK (52, 54). AMPK-mediated activation of nitric oxide synthase 3 (NOS3/eNOS) leads to enhanced nitric oxide (NO) levels and NO-derived reactive nitrogen species, contributing to PTEN SNO on Cys-83 (52, 53, 55). This modification is important for the proliferation of PTEN proficient cells under energy-deprived conditions (52). Of note, the AKT kinase can also signal directly to activate eNOS, contributing to high NO levels (56). Nitrosative stress-induced posttranslational modifications, unlike in the case of PTEN, are not always limited to inhibitory effects, and can result in enhanced stabilization and activation of oncogenes including EGFR and Src that also contribute to the activation of the PI3K/Akt pathway (57). Taken together, these findings suggest a model whereby multiple oncogenic signaling cascades, in particular PI3K/Akt signaling, can be potentiated through elevated nitrosative stress to promote pro-survival adaptations during nutrient deprivation.

Reactive oxygen species can also modulate PI3K/Akt signaling by regulating protein tyrosine phosphatases (PTPs), which inhibit RTKs such as EGFR and PDGFR through dephosphorylation. PTPs can be broadly classified into four main groups based on their specific substrates and all contain an essential cysteine residue in the catalytic domain (58). ROS—and H_2_O_2_ in particular—have been shown to reversibly oxidize this cysteine residue, leading to reduced phosphatase activity of PTPs and sustained RTK activation (59). Notably, detoxification of H_2_O_2_ by overexpressing catalase or inhibition of NOXs contributes to a marked reduction in tyrosine phosphorylation of PDGFR, EGFR, and the insulin receptor (60, 61). Perhaps the most potent activator of PI3K/Akt signaling is insulin, and stimulation of IRS-1 following insulin binding, increases H_2_O_2_ generation and NOX4 activity (62). PTP1B and SHP-2 are the main PTPs that dephosphorylate IRS-1; therefore, their inhibition in response to insulin-induced ROS levels facilitates persistent activation of many downstream signaling cascades including the PI3K/Akt pathway (62). The implications of this regulation in the cancer context are not only limited to increased proliferation and metastatic potential, but also play a major role in promoting drug resistance (63, 64). The development of drug resistance is particularly relevant in breast cancer, where dysregulated IRS-1/IGF-1R signaling decreases the sensitivity to tamoxifen and trastuzumab in estrogen receptor (ER)- and HER2-positive tumors, respectively, by potentiating ERK and PI3K/Akt signaling (63, 65, 66). Thus, by inhibiting several key negative regulators, redox stress has a significant role in the activation of PI3K/Akt signaling and associated pro-tumorigenic phenotypes.

## PI3K/AKT Signaling Maintains Redox Balance in Cancer Cells

**Figure 2:**
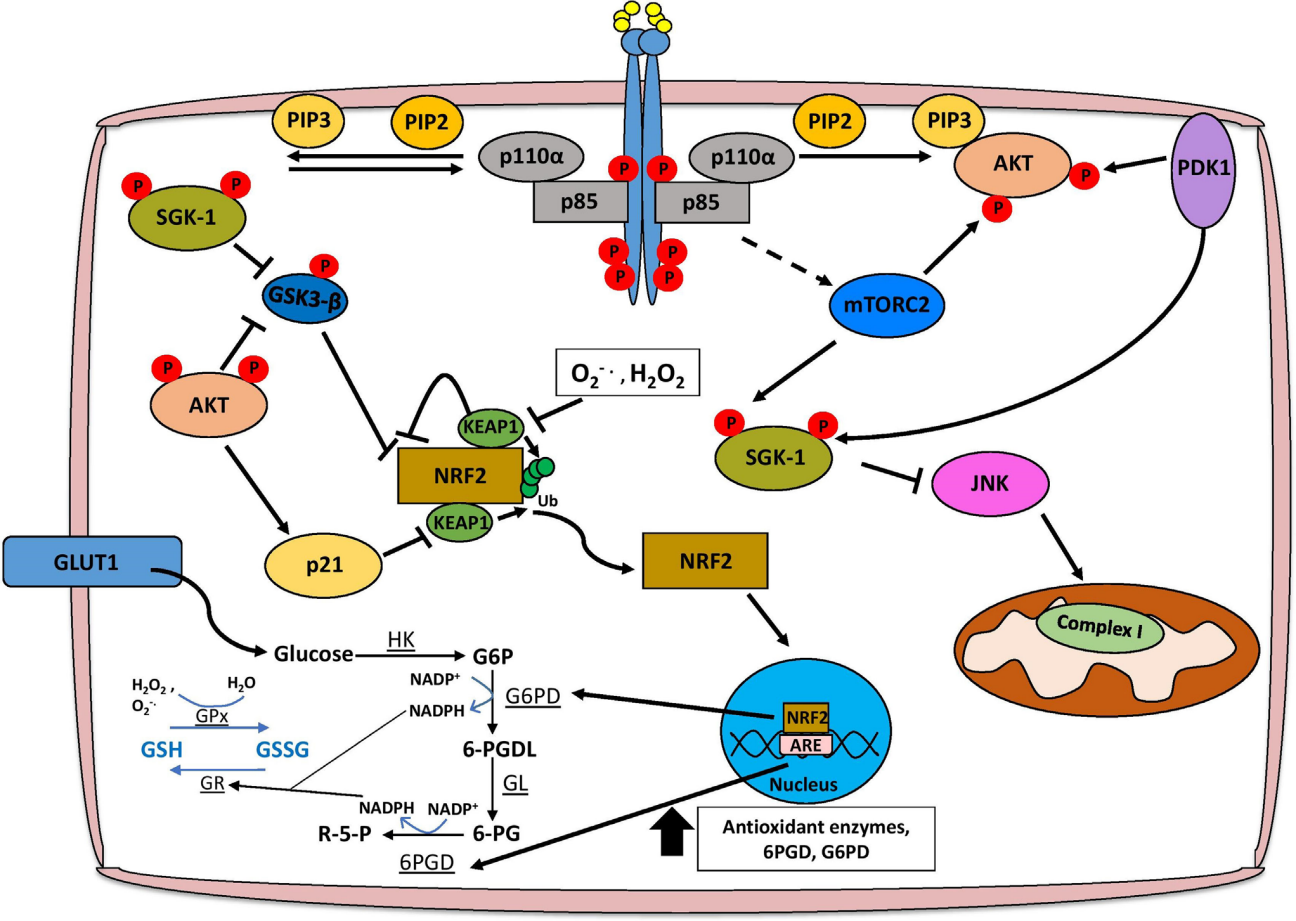
PI3K/Akt signaling facilitates ROS detoxification and redox homeostasis. The concomitant increase in the cellular antioxidant response is necessary for redox homeostasis, and this is largely mediated through NRF2 signaling, glutathione metabolism, and production of NADPH. AKT facilitates the activation of NRF2 by inhibiting the interaction with KEAP1 and alleviating the negative regulation imposed by GSK3-β. Functional NRF2 translocates to the nucleus and transcriptionally activates genes involved in the antioxidant response such as glutathione S-transferase and superoxide dismutase, as well as the pentose phosphate pathway (PPP), which produces NADPH. Glutathione biosynthesis is an important antioxidant, which is regulated in a PI3K/Akt/Nrf2-dependent mechanism, and the conversion of glutathione between reduced (GSH) and oxidized (GSSG) forms is dependent on PPP-derived NADPH. Finally, Akt-independent signaling axes through SGK-1 may also promote ROS detoxification. Abbreviations: HK, hexokinase; G6P, glucose-6-phosphate; G6PD, glucose-6-phosphate dehydrogenase; 6-PGDL, 6-phosphogluconolactone; GL, gluconolactonase; 6-PG, 6-phosphogluconate; 6-PGD, 6-phosphogluconate dehydrogenase; R-5-P, ribulose-5-phosphate; GR, glutathione reductase; GPx, glutathione peroxidase; SGK, serum and glucocorticoid-regulated kinase; PI3K, phosphoinositide 3-kinase.

In order for ROS to confer a selective advantage to cancer cells, there must be a concomitant increase in antioxidant responses to prevent adverse effects on cell viability. These responses include upregulation of the Keap1–Nrf2 pathway and modulation of glutathione metabolism, both of which are regulated by PI3K/Akt signaling (Figure 2). The transcription factor Nrf2 is recognized as a central mediator of ROS detoxification by inducing the expression of several enzymes that are involved in the antioxidant response, including glutathione *S*-transferase, superoxide dismutases, and NAD(P)H:quinone oxidoreductase 1 (Nqo1) (67). Under low concentrations of cellular ROS, Nrf2 is bound to the E3 ubiquitin ligase Keap1 in the cytosol and degraded by the proteasome (68). This interaction is inhibited under high concentrations of ROS, which oxidize cysteine residues on Keap1, thus allowing Nrf2 to translocate to the nucleus and induce the expression of genes containing antioxidant response elements (69). PI3K/Akt activation has been shown to be essential for the nuclear translocation of Nrf2, and accordingly treatment of neuroblastoma SH-SY5Y cells with PI3K inhibitors LY294002 and wortmannin, but not MAPK inhibitors, reduces Nrf2 transcriptional activation of antioxidant genes (70). One of the most important implications of PI3K-mediated upregulation of Nrf2 signaling is promoting cancer cell survival by conferring protection against excessive oxidative stress (71, 72). This is particularly relevant in BRCA1-deficient breast cancers, which lack effective DNA repair mechanisms through homologous recombination and are therefore susceptible to genetic modification induced by ROS (73). Interestingly, although up to 80% of BRCA1 mutant breast cancers are ER negative, estrogen positively regulates Nrf2 transcriptional activity through activation of PI3K/Akt signaling allowing BRCA1-null cells to detoxify high ROS levels and accumulate additional genetic aberrations that may contribute to tumorigenesis (73, 74). Inhibition of PI3K/Akt signaling either by PTEN overexpression or BKM120 treatment hinders estrogen-mediated Nrf2 activation, suggesting that targeting this pathway might be beneficial in treating BRCA1-deficient tumors by re-sensitizing them to elevated ROS levels (73).

Phosphoinositide 3-kinase/Akt activation has both Nrf2-dependent and -independent effects on cellular metabolism that contribute to ROS detoxification. Several antioxidant pathways rely on the reducing power of NADPH, which is predominantly generated by 6-phosphogluconate dehydrogenase (6PGD) and glucose-6-phosphate dehydrogenase (G6PD) from the pentose phosphate pathway (PPP) (75, 76). Active Nrf2 induces the expression of the aforementioned PPP enzymes through an AKT-dependent mechanism, as well as enzymes directly involved in NADPH synthesis such as malic enzyme 1 (ME1) and isocitrate dehydrogenase IDH (77). As AKT is a potent activator of glucose uptake and glycolysis, tumors dependent on PI3K/Akt signaling could shunt the glucose-6-phosphate (G6P) generated during glycolysis to the PPP activated by Nrf2, thus maintaining a stable pool of NADPH, which could be used for anabolic processes to sustain tumor growth and proliferation, or ROS detoxification (77, 78) (Figure 2).

Phosphoinositide 3-kinase/Akt and Nrf2 signaling, as well as the associated increase in NADPH are key regulators of the synthesis of glutathione, which exists in either reduced (GSH) or oxidized (GSSG) form (79). Under conditions of oxidative stress, enzymes such as GSH transferase (GSH-Tr) and GSH peroxidase (GPx) facilitate the reduction of ROS, including H_2_O_2_, by oxidizing GSH to GSSG (79). In order to completely detoxify ROS, oxidized GSSG must then be reduced back to GSH by NADPH-dependent glutathione reductase (GSSG-Rx) (79). The induction of glutathione synthesis through upregulation of glutamate-l-cysteine ligase, as well as transcriptional activation of GSH-Tr and GPx is dependent on a PI3K/Akt/Nrf2 signaling axis (80, 81). AKT can also increase the stability of Nrf2 by activating p21Cip1/WAF1 which disrupts the interaction between Keap1 and Nrf2, and by inhibiting GSK-3β that leads to the reduction of Nrf2 phosphorylation, preventing its nuclear export and ubiquitination (82, 83). Importantly, glutathione biosynthesis has been shown to be a metabolic vulnerability in *PIK3CA* mutant breast cancer cells, as treatment of *PIK3CA* or *AKT* mutant MCF10A cells with buthionine sulfoximine (BSO) significantly reduces anchorage-independent growth and inhibits cell proliferation in 3D, but not 2D culture (83). Furthermore, cisplatin treatment when given in combination with BSO leads to tumor regression of *PIK3CA* mutant, but not wild type, cell line-derived xenograft models indicating that disrupting redox homeostasis through GSH metabolism could repurpose existing therapies for the treatment of *PIK3CA* mutant breast cancers (83).

Although AKT is perhaps the most extensively characterized downstream effector of PI3K signaling, recent studies have highlighted the importance of an Akt-independent axis which relies on other members of the AGC serine/threonine kinase family such as PDK1, RSK, and SGK1 (84, 85). In particular, SGK1 has been shown to promote antioxidant responses during pregnancy that are essential for fetal development, and exert protective effects in Parkinson’s disease (86). SGK1 can negatively regulate JNK signaling, an important inducer of superoxide species by modulating complex I activity of the electron transport chain (87, 88). In addition, SGK1-mediated inactivation of GSK3-β facilitates MCL1 localization to the mitochondria to significantly reduce mitochondrial ROS production through inhibition of NOX4 expression, adversely affecting the response to chemotherapy (88, 89). It is important to note, however, that in models of lung cancer, MCL1 may also promote ROS production from the mitochondria by binding to voltage-dependent anion channels and increasing the mitochondrial flux of calcium (90).

## Therapeutically Exploiting Redox Homeostasis in PI3K-Dependent Tumors

In terms of redox homeostasis, it is clear that PI3K/Akt signaling is unique in that it activates both ROS generating and detoxifying processes, thus creating a stable presence of ROS, which can exert pro-tumorigenic effects. This raises the attractive therapeutic prospect of perturbing redox homeostasis in tumors with hyperactive PI3K/Akt signaling either alone or in combination with existing treatments. Chemotherapy and radiotherapy are among the most common therapeutic interventions for cancer patients, and one mechanism through which they induce apoptosis in cancer cells is by upregulating ROS (91). Anthracyclins, such as doxorubicin, and platinum-based therapies including cisplatin, can directly induce ROS production through modulation of the electron transport chain, and elevated ROS levels subsequently activate caspases, cytochrome *c* release, and DNA damage leading to apoptosis (92, 93). Previous clinical studies have demonstrated that *PIK3CA* mutant breast cancers display decreased sensitivity to anthracyclines and cisplatin, however, as these drugs exert anti-cancer effects through ROS upregulation, it is conceivable that the antioxidant pathways, which are elevated by PI3K/Akt signaling, could counteract these therapies (91, 94). Indeed, active Nrf2 protects cancer cells from ROS induced cell death, and inhibition of this transcription factor by brusatol treatment enhances the response to cisplatin (95, 96). In addition, targeting glutathione biosynthesis with BSO selectively sensitizes *PIK3CA* mutant breast tumors to cisplatin as compared to wild-type ones, indicating that impairing the antioxidant response could be an exploitable vulnerability in PI3K-driven tumors (83).

The apparent anti-cancer effects of excessive ROS accumulation are also important in targeting tumors, which have become resistant to PI3K/Akt inhibitors. A common strategy to overcome resistance is to place patients on “drug holidays” and subsequently re-challenge them with the inhibitor (97). In PI3K inhibitor resistant breast cancer cells, substantial metabolic rewiring occurs following removal of the class IA PI3K inhibitor GDC-0941 that is characterized by increased glycolysis and mitochondrial respiration (98). During drug holidays, the metabolic phenotype of resistant cancer cells is altered by an Akt-independent PI3K/mTORC1 signaling axis, which drives excessive production of ROS and inhibits the proliferation of these cells (98). Notably, this proliferative defect is rescued following treatment of resistant cells with ROS scavengers such as NAC, thus demonstrating the importance of regulating ROS homeostasis in prohibiting the expansion of a resistant cell population emerging through therapies (98). These findings present an opportunity to improve responses by specifically exploiting the unique metabolic profile of PI3K inhibitor resistant cells, either through glucose deprivation or by further increasing oxidative stress (98). Developing resistance to AKT-specific inhibitors such as MK2206 is also a significant clinical problem, and studies into the identification of synthetic lethal interactions between AKT and antioxidant inhibitors have shown promising results in overcoming drug resistance (99). In non-small cell lung carcinoma (NSCLC) models, dual treatment with the thioredoxin reductase-1 (TXNRD1) antioxidant inhibitor auranofin and MK2206 induced cancer cell-specific apoptosis through ROS-stimulated JNK signaling (99). Importantly, this synthetic lethality was observed in lung tumors with functional NRF2–KEAP1 signaling and overexpression of TXNRD1, indicating that the activity of the anti-oxidative response in NSCLC could be used as a biomarker for determining which patients may benefit from dual AKT/TXNRD1 inhibition (99).

Although it seems that enhancing ROS production may have inhibitory effects on cancer cells, it is important to note that finding the balance between antioxidant and ROS-generating mechanisms is complex and so is their therapeutic exploitation (100). Notably, there is still significant debate regarding the administration of antioxidants during cancer therapy as a means of limiting drug toxicity and whether their use adversely affects the patient’s response, and/or the potential systemic consequences of deliberately elevating ROS levels (101). It is, therefore, necessary to consider all of these implications and to ensure that tumors are well characterized at the genetic and metabolic levels to determine if targeting redox homeostasis is a suitable treatment option.

## Conclusion and Future Perspectives

Several pro-tumorigenic processes converge on hyperactive PI3K/Akt signaling, and it is becoming increasingly evident that ROS metabolism is no exception to this. The capacity for the PI3K/Akt cascade to directly activate both ROS generating and various antioxidant pathways suggests a tight regulation on cellular redox homeostasis, the intricacies of which merit further investigation. Understanding the complex interplay between ROS and PI3K/Akt signaling is particularly relevant for developing therapeutic strategies to target tumors dependent on this pathway, especially since recent clinical trials have demonstrated only modest responses to PI3K/Akt pathway inhibitors and development of resistance (102). While ROS metabolism certainly adds another layer of complexity to PI3K/Akt signaling, this area of research holds great promise, not only for the potential identification of novel biomarkers and metabolic dependencies but also the prospect of implementing more potent therapeutic combinations, which perturb redox homeostasis and effectively target PI3K-driven tumors.

## Author Contributions

NK and GP wrote the manuscript and designed the figures.

## Conflict of Interest Statement

The authors declare that the research was conducted in the absence of any commercial or financial relationships that could be construed as a potential conflict of interest.
